# Factors Affecting the Length of Stay in the Intensive Care Unit: Our Clinical Experience

**DOI:** 10.1155/2018/9438046

**Published:** 2018-03-20

**Authors:** Mehmet Toptas, Nilay Sengul Samanci, İbrahim Akkoc, Esma Yucetas, Egemen Cebeci, Oznur Sen, Mehmet Mustafa Can, Savas Ozturk

**Affiliations:** ^1^Department of Anesthesiology, Haseki Training and Research Hospital, Istanbul, Turkey; ^2^Department of Internal Medicine, Haseki Training and Research Hospital, Istanbul, Turkey; ^3^Department of Biochemistry, Haseki Training and Research Hospital, Istanbul, Turkey; ^4^Department of Nephrology, Haseki Training and Research Hospital, Istanbul, Turkey; ^5^Department of Cardiology, Haseki Training and Research Hospital, Istanbul, Turkey

## Abstract

**Background and Aim:**

Long hospital days in intensive care unit (ICU) due to life-threatening diseases are increasing in the world. The primary goal in ICU is to decrease length of stay in order to improve the quality of medical care and reduce cost. The aim of our study is to identify and categorize the factors associated with prolonged stays in ICU.

**Materials and Method:**

We retrospectively analyzed 3925 patients. We obtained the patients' demographic, clinical, diagnostic, and physiologic variables; mortality; lengths of stay by examining the intensive care unit database records.

**Results:**

The mean age of the study was 61.6 ± 18.9 years. The average length of stay in intensive care unit was 10.2 ± 25.2 days. The most common cause of hospitalization was because of multiple diseases (19.5%). The length of stay was positively correlated with urea, creatinine, and sodium. It was negatively correlated with uric acid and hematocrit levels. Length of stay was significantly higher in patients not operated on than in patients operated on (*p* < 0.001).

**Conclusion:**

Our study showed a significantly increased length of stay in patients with cardiovascular system diseases, multiple diseases, nervous system diseases, and cerebrovascular diseases. Moreover we showed that when urea, creatinine, and sodium values increase, in parallel the length of stay increases.

## 1. Introduction

Intensive care units (ICU) are high tech special treatment units that developed for close monitoring, rapid intervention, followup, and treatment of acute disease [1]. Life-threatening organ failure of chronic disease demands for intensive care beds are increasing in the world and in our country because of increasing general and aging population. We should select patients carefully because of expensive treatment and limited number of intensive care beds. Long hospital days in the intensive care units cause high costs and affect patients, their families, and the country's economy. And also longer length of stay in the ICU affects mortality. In order to improve the quality of medical care, the primary goal in intensive care is to decrease length of stay and reduce cost [2].

In our country studies about factors affecting length of stay in the intensive care units are limited. So our aim is to detect the factors associated with prolonged stays in the intensive care.

## 2. Material and Methods

### 2.1. Patients

In this study we retrospectively analyzed 3925 patients admitted between January 2008 and December 2014 in Haseki ICU. We obtained the patient demographic, clinical, diagnostic, and physiologic variables, mortality, and lengths of stay by examining the intensive care unit database records. The study was approved by the Ethical Committee of the Haseki training and research hospital and written informed consent was obtained. Exclusion criteria included patients younger than 18 years. Data input is performed by intensive care specialist.

### 2.2. Statistical Analysis

Statistical analyses were performed using the SPSS software (version 16.0, SPSS, Chicago. Ill). Descriptive statistics are summarized as frequencies and percentages for categorical variables and as the mean, standard deviation, minimum, and maximum for continuous variables. Mann–Whitney *U* test was used to compare differences between two independent groups because numeric variables do not have a normal distribution. Because parametric test condition is not provided, relationships between quantitative variables were examined with Spearman Correlation Analysis. Kruskal–Wallis test was performed for groups more than two. Statistical significance was accepted at a *p* value less than 0.05. Determinant factors were investigated by Linear Regression Analysis Forward Method.

## 3. Results

2185 men and 1741 women were included in the total of 3925 patients who underwent hospitalization in intensive care unit. The mean age of the study was 61.6 ± 18.9 years (median = 65 years). The average length of stay in intensive care unit was 10.2 ± 25.2 days. The median length of stay was 2 days and ranges between quarters were 1–7 days. 48.5% of patients were operated on. The mortality rate was 32.5% (Table 1).

The reason of hospitalization was because of 19.5% because of multiple diseases (diabetes mellitus, hypertension, vasculitis, etc.), 16.8% cerebrovascular diseases, 13% gastrointestinal diseases, 9.5% respiratory disease, cardiovascular diseases 9%, 8.7% urogenital diseases, 6.8% musculoskeletal diseases, 4.9% hepatobiliary diseases, and 3.7% endocrine diseases. Biochemical parameters are summarized in Table 2.

The length of stay was positively correlated with urea, creatinine, and sodium. It was negatively correlated with uric acid and hematocrit levels (*p* < 0.001, rho: 0.180; *p* < 0.001, rho: 0.154; *p* = 0.002, rho: 0.049; *p* = 0.024, rho: −0.282; *p* = 0.048, rho: −0.032) (Table 3). There was no statistically significant difference between sexes (*p* = 0.873). ICU stay day was significantly higher in patients not operated on than in patients operated on (*p* < 0.001). The stay day in ICU was longer in patients with multiple diseases (16.1 ± 30.4, *p* < 0.001).

In univariate analysis in models that are created from variables of *p* < 0.100, urea, AST, sodium levels, cardiovascular system diseases, multiple diseases, nervous system diseases, and cerebrovascular diseases were identified as factors that determine the length of stay (Table 4).

## 4. Discussion

The characteristics of prolonged ICU stay would be useful, if some factors could be modified. These factors should include process of care, active relevance of ICU physicians, and length of hospital stay before ICU admission. And so patients with long length of stay and thus high costs can be identified early [3, 4].

In intensive care units to measure disease severity they use many scoring systems consisting of various parameters. APACHE II is one of this scoring systems [5]. APACHE II scoring system is considered to show good correlation with the risk of mortality and hospital-acquired infections [6]. We did not use APACHE scoring systems because our aim is not to predict the mortality; in this study we want to predict prolonged length of stay.

Our study showed a significantly increased length of stay in patients with cardiovascular system diseases, multiple diseases, nervous system diseases, and cerebrovascular diseases. In a prospective study by Wong et al., for patients in ICU, the most common reasons for admission were neuromuscular weakness, pneumonia, multiple traumas, and septic shock, in this order. Respiratory arrest, cardiac arrest, congestive heart failure, postoperative mechanical ventilation, airway protection or obstruction, and chronic obstructive pulmonary disease were the next most common indications for ICU admission in these patients [7].

And in one study, postoperative patients' length of stay in ICU was shorter than that of patients admitted to the ICU for other reasons [8]. Similarly our study showed that length of stay was significantly higher in patients not operated on than in patients operated on.

Making changes in any of the medical factors about illness which affect length of stay needs expert medical skills [9, 10]. And also psychological, social, and institutional factors have effect on ICU length of stay [2, 11] Ahrens et al. found that when a specialized team consisting of a physician and a clinical nurse specialist works in ICU, the length of stay would be shorter [12]. But we did not study this area.

Our study showed that when urea, creatinine, and sodium values increase, in parallel the length of stay increases. This means that physicians should pay attention to kidney injury and rehydration.

Also there are social and psychological factors that affect the length of stay, but we want to emphasize the medical factors.

## 5. Conclusion

Our study showed a significantly increased length of stay in patients with cardiovascular system diseases, multiple diseases, nervous system diseases, and cerebrovascular diseases. Moreover we showed that when urea, creatinine, and sodium values increase, in parallel the length of stay increases. Further studies could supply a strategy for targeting the specific risk factors.

## Figures and Tables

**Table 1:** 

	Mean ± SD
Age	61.6 ± 18.9 (1–101)
Sex	
Men	2185 (55.7)
Women	1740 (44.3)
Operation	
None	2021 (51.5)
Existing	1904 (48.5)
Length of stay	10.2 ± 25.3 (0–418)
Result	
Still alive	2648 (67.5)
Exitus	1277 (32.5)

**Table 2:** 

	Mean ± SD	Min	Max
Urea	64.0 ± 56.4	5	565
Creatinine	1.7 ± 2.0	0.1	25.7
Uric acid	5.4 ± 3.0	1	13
Albumin	2.7 ± 0.7	1	9
LDH	519.0 ± 1074.7	3	20540
AST	158.0 ± 591.1	0	9657
ALT	116.5 ± 338.7	1	3666
Sodium	138.1 ± 6.2	89	192
Hematocrit	33.4 ± 7.1	7	83
Hemoglobin	11.0 ± 2.4	2	25
Platelet	239.2 ± 130.4	2	3258
WBC	13.5 ± 8.6	0	170

LDH: lactate dehydrogenase; AST: aspartate dehydrogenase; ALT: alanine dehydrogenase; WBC: white blood cells.

**Table 3:** 

	Length of stay	
	rho	*p*
Age	0.026	0.107
Urea	0.180	<0.001
Creatinine	0.154	<0.001
Uric acid	−0.282	0.024
Albumin	0.014	0.568
LDH	−0.022	0.396
AST	−0.040	0.076
ALT	0.023	0.373
Sodium	0.049	0.002
Hematocrit	−0.032	0.048
Hemoglobin	−0.028	0.087
Platelet	0.003	0.864
WBC	0.011	0.498

LDH: lactate dehydrogenase; AST: aspartate dehydrogenase; ALT: alanine dehydrogenase; WBC: white blood cells.

**Table 4:** 

	*B*	Beta	*p*
Constant value	−16,355		
Operation	−9,925	−0,134	<0,001
Urea	−0,046	−0,087	<0,001
Sodium	0,278	0,059	0,014
AST	−0,003	−0,052	0,029
Cardiovascular system	9,286	0,081	0,002
Multiple disease	6,107	0,078	0,004
Cerebrovascular disease	9,302	0,103	<0,001

AST: aspartate dehydrogenase. Uric acid was not included in the model because of insufficient number.
